# Challenges

**DOI:** 10.1530/EOR-2024-0200

**Published:** 2025-01-03

**Authors:** Pierre Hoffmeyer

**Affiliations:** Editor-in-Chief, *EFORT Open Reviews*

Orthopaedics has come a long way, from prolonged bed rest, transosseous traction and body casts. We have witnessed the advent of instantaneous communication, modern imagery, performing prosthetics and mini-invasive surgery. Now, we face the tsunami of artificial intelligence and instantaneous communication amongst other contemporary progresses. Even though much of our practice has evolved beyond many a baby boomers’ dream, there remain challenges yet to be conquered by this and future generations.

We have definitely not solved infection. This continues to plague orthopaedic practice, and few answers are on the horizon. Iodine skin prep, clean air, space suits and antibiotic prophylaxis have improved the statistics. Progress continues, and today’s push for the early administration of oral antibiotics for treating osseous infections diminishes hospitalization and increases patient comfort. Percentage numbers of patients acquiring post-surgical infections are steadily decreasing. Nevertheless, percentage numbers, however small, represent individual patients and zero post-operative infections remains a distant goal. Progress has been made in the care of patients victims of infections, but headway is slow. Bacteria have found ways to becoming resistant to even the most powerful antibiotics. Glycocalyx still represents an insurmountable barrier for the penetration of standard antibiotic therapy. Patients with open fractures caused by high-power weapons in war zones are plagued with bacteria resistant to all known antibiotics, and amputation is for many the only solution. New avenues include antibiotic-laden implants, nanotechnology and phage therapy.

Implant wear still represents a major challenge. Although the days of vanishing bone disease due to particulate release linked to standard polyethylene use are long gone, nonetheless problems remain. Registry data tend to show that today’s implants around the hip and the knee are expected to last two decades before wear sets in and reoperation becomes necessary. The metal-on-metal disaster has not solved the issue of longevity although some of these implants having passed the ten-year mark have shown to be long-lasting. Ceramic on ceramic might be a solution, but breakage, squeaking and high costs are a major hindrance. Highly cross-linked polyethylene produced by irradiation or chemically with antioxidant additives has prolonged the implant life at least in the hip. For the knee, controversies remain as to which polyethylene type is the most suited. Enhanced highly cross-linked polyethylene seems to be the best bet today for minimizing wear and providing long life. Given that research and testing are ongoing in materials science, we still require implants guaranteed to last the lifetime of the patient notwithstanding age or activity level.

Bone heals, yet no one has come up with an accelerator fracture union. From the era of the dinosaurs to our 21st century, adult bone takes 3–4 months to unite. Nature has put no ‘effort’ into decreasing healing time to union. Most animal species are long dead, eaten or decaying before a fractured limb occurring in the wild has time to unite. The only exceptions are the young, cared for, nurtured and fed by the mother. Compare that to the 1/1000th of a second it takes for initiating the coagulation cascade, ensuring survival in the face of a minor scratch. Our fracture implants stabilize the skeleton, but faster healing is still a utopian dream. Drugs such as bisphosphonates and parathyroid hormone have only a meagre effect on bone healing. Although many avenues are explored, such as PRP, nanoparticles and hormonal therapy, up to the present, there have been only marginal results. The same is true for ligaments and cartilage, which are, at best, replaced by inefficient scar tissue. Progress is wanted in the field of enhancing and speeding tissue repair.

Orthopaedic surgeons also need to recognize the burden that trauma-related psychological distress inflicts on our patients. Too often, this goes undetected, minimized or brushed over, leading to poor results occurring even in the most innocuous injury. Obviously, dealing with severe posttraumatic psychic trauma is not within the realm of expertise of orthopaedic surgeons; however, these complex issues need recognition so that the patients may be referred, in a timely matter, to the appropriate specialist. Patients need support in this area by their surgeon, thereby ensuring a rapid recovery and return to work or sports.

Musculoskeletal diseases are the leading cause of years lived with disability. This entails high costs and involves the activities of orthopaedic surgeons. Healthcare systems all over the world are overstretched and beleaguered by financial difficulties. Orthopaedic surgeons must be aware at all times that the inappropriate use of scarce resources will result in restrictive regulatory measures detrimental not only to our practices but also to our patients. Although representing sometimes painful decisions; efficiency must be the guide to our practices.

These are some of the challenges facing us as a profession, and we must support research efforts in order to advance solutions and perhaps even solve some of these issues, hopefully in the not too distant future. For the practitioner, to keep abreast of developments in these fields is of vital importance.

*EFORT Open Reviews* thanks its readers for their fidelity and wishes all a knowledgeable and fruitful New Year.

